# Supplementary material for: Sex-dependent and -independent transcriptional changes during haploid phase gametogenesis in the sugar kelp Saccharina latissima
Source: PLoS One. 2019 Sep 12;14(9):e0219723. doi: 10.1371/journal.pone.0219723 (PMC6742357; doi:10.1371/journal.pone.0219723)
Supplement: S1 File — A. Alignment in nexus format of 38 INO1 amino acid sequences obtained after BLASTP analysis (NCBI nr) of 2 INO1 predicted proteins identified in the S. latissima transcriptome (SL_25748.1_384_1991_- and SL_25960.2_333_2118_-). The alignment was performed using Muscle[1] and curated with Gblocks[2] and low stringency parameters to remove gaps and poorly-aligned regions, resulting in 486 amino acid characters. B. ML phylogenetic tree of stramenopile INO1 protein sequences. The tree was built using PhyML[3] (LG model; aLRT branch support; model-given amino-acid frequencies; optimized across-site rate variation; best of NNI and SPR tree search). S. latissima INO1 contigs are well-supported within the Phaeophyceae (brown algal) clade. The non sex-biased copy SL_25748.1 is sister to the S. japonica INO1. The female-biased copy SL_25960.2 is more divergent. It should be noted that following Lipinska et al. (2017)[4], the S. japonica genome sequence derives from a male gametophyte strain. (DOCX) [file pone.0219723.s001.docx]

**S1 File.** Alignment and phylogenetic analysis of Myo-inositol-1-phosphate synthase (INO1) predicted proteins from stramenopiles.

1. Alignment in nexus format of 38 INO1 amino acid sequences obtained after BLASTP analysis (NCBI nr) of 2 INO1 predicted proteins identified in the *S. latissima* transcriptome (SL_25748.1_384_1991_- and SL_25960.2_333_2118_-). The alignment was performed using Muscle^[1]^ and curated with Gblocks^[2]^ and low stringency parameters to remove gaps and poorly-aligned regions, resulting in 486 amino acid characters.

#NEXUS

BEGIN DATA;

DIMENSIONS NTAX=38 NCHAR=486;

FORMAT DATATYPE=PROTEIN

GAP=-

;

MATRIX

[1] 'SL_25748.1_384_1991_-'

VQSPDVTYTDDEMVSKYTYQTTLVIVTPVSQTLHFKTQTKVPRVGVMLVGLGGNNGSTVA

AGILANQNGLTWNTKEGEKKANYWGSLCMASTVRLGNDAAGNAVHVPLRNMLPTVHPNDL

VLGGWDICSDNLADAMGKASVLDFDLQQKLKPMMVDMVPLPSIYFPDFIAANQSERADNV

LKGSKQEQMDQIRKDIREFKEKNKLDKVVVLWTANTERFSALEAGINDTKDNILASITRG

EEEVSPSTIFACASILEGCSYINGSPQNTFVPGVVELAEEHKVYIGGDDFKSGQTKMKSV

MVDFLVSAGIKPTSIVSYNHLGNNDGRNLSAPQQFRSKEISKSNVVDDMVASNRLLYKAG

EHPDHVVVIKYVPAVKDSKRALDEYTSEIFMGGLNTISMHNTCEDSLLASPLIFDLVIMA

ELCERIQVG-GKWESFHSVLSLLSYMLKAPLVPRGTPVVNALFAQRQAIINVMRACGLAP

ENHMLL

[2] 'SL_25960.2_333_2118_-'

VASPNVTYTAETITSNYEYRTTDVVATPRVQTYQFKTQRVVPRTGVMIVGWGGNNGTTVT

GGVIANREGISWNTKAGVRNPNYFGSITQASTINVGS-TGGKDVFVPFSSVLPMVNPNDL

VIGGWDINNMNLGDAMARAEVFHIDLQRKLYPHMKEMKPLPSVYVPDFIAANQSERADNV

LKGSKQEQMDQIRKDIREFKEKNKLDKVVVLWTANTERFSALEAGINDTKDNILASITRG

EEEVSPSTIFACASILEGCSYINGSPQNTFVPGVVELAEEHKVYIGGDDFKSGQTKMKSV

MVDFLVSAGIKPTSIVSYNHLGNNDGRNLSAPQQFRSKEISKSNVVDDMVASNRLLYKAG

EHPDHVVVIKYVPAVKDSKRALDEYTSEIFMGGLNTISMHNTCEDSLLAAPLILDLALLC

ELCERITVAAGATTKFHSVLSLLSYLLKAPMVPPGTPVVNALFKQREAIVNVMRACGLAP

ENFMAL

[3] SJ17716

------------------------------------------------------------

------------------------------------------------------------

--------------------------------MMVDMVPLPSIYFPDFIAANQSERADNV

LKGSKQEQMDQIRKDIREFKEKNKLDKVVVLWTANTERFSALEAGINDTKDNILASITRG

EEEVSPSTIFACASILEGCSYINGSPQNTFVPGVVELAEEHKVYIGGDDFKSGQTKMKSV

MVDFLVSAGIKPTSIVSYNHLGNNDGRNLSAPQQFRSKEISKSNVVDDMVASNRMLYDED

EHPDHV------------------------------------------------------

------------------------------------------------------------

------

[4] CBN77493.1

VRSPDVTYTDDEMVSKYTYQTTLVVVTPKSETLYFKTQTKVPKVGVMLVGLGGNNGSTVA

AGILANKHGLTWNTKEGEKKANYWGSLCMASTVRLGNDSNGNAVNVPLRNLLPTAHPNDF

VLGGWDICSDNLADAMVKASVLDYDLQQKLKPMMVDKKPLPSIYFPDFIAANQSERADNV

LTGTKQEQMDQIRKDIREFKEKNNLDKVVVLWTANTERFSALEPGINDTKENILASITRG

EEEVSPSTIFACASILEGCSYINGSPQNTFVPGVIELAEEHKVYIGGDDFKSGQTKMKSV

MVDFLVSAGIKPTSIVSYNHLGNNDGRNLSAPQQFRSKEISKSNVVDDMVDSNKMLYKEG

EHPDHVVVIKYVPYVGDSKRALDEYTSEIFMGGKNTISMHNTCEDSLLASPLIFDLVIMA

ELCERIQVG-GKWEGFHSVLSLLSYMLKAPLVPPGTPVVNALFAQRQAIINVMRACGLAP

ENHMLL

[5] XP_002292968.1

VESDSCKYTDDAIIADFSYDTTVVTVKPSSTKMQFKTERKVGKTGVMLVGLGGNNGCTCV

AGAMANKLGLTWETKEGTQKANYWGSLLMASTVKLGNDRHGNSVYTPMSNMLPLLNPNDI

VWGGWDINGTNLGDAMKRSKVLDYDLQQKLYPHMKDITPLPSIYFPDFIAANQGERADNI

LTGTKQEQMDQIRKDIKDFKMKNGLDQVILLWTANTERFSAVEEGVNDTAENILSSIEMG

EAEVSASTVFAVASILEGCTFINGSPQNTFVPGVIELARQKKVFIAGDDFKSGQTKMKSV

MVDFLVSAGIKPVSIVSYNHLGNNDGKNLSAPSQFRSKEISKSNVVDDMVASNRILFEED

EHPDHVVVIKYVPYVADSKRAMDEYTSEIFMGGKNTIVMHNTCEDSLLATPLIYDLVILG

ELCARVQVGESEWESFHPVLSLLSYMLKAPLVPNGAPVVNALFTQRCAIVNFMRACGLAP

DNHMTL

[6] EJK66432.1

VESDSVTYTDEAIISDYSYETTLVTVKPVTTKMQFKTERNVGQTGVMLVGMGGNNGTTCI

AGAIANKLGLTWETKDGTQKANYFGSLLLASTVKLGNDPQGNSVYTPMNNMLPLLNPNDI

VWGGWDINGANLGDAMKRAKVLDHDLQTKLYPHMKDITPLPSIYFPEFIAANQSERADNI

LKGTKQEQLEQVRKDIRDFKREHGLDQVIVLWTANTERFSSVDKGINDTAENLIRSIDMG

ESEVSASTIFAVASILEGSTFINGSPQNTFVPGVIDLARQEKVFIAGDDFKSGQTKMKSV

MVDFLVSAGIKPTSIVSYNHLGNNDGKNLSAPSQFRSKEISKSNVVDDMVASNRILFEEN

ERPDHVVVIKYVPYVGDSKRAMDEYTSEIFMGGKNTIVMHNTCEDSLLATPLIYDLVILG

DLCKRVQIDESEWESFHPVLSLLSYMLKAPLVPNGAPVVNALFTQRCAVINFMRACGLAP

DNHMTL

[7] GAX27321.1

VQSSKCQYKSDAIIANFNYETTLVVVRPVETKLQFRTERKVPKLGVMLVGMGGNNGCTCI

AGAIANKLKLTWQTKEGEKKANYWGSVMLASTVKLGNDAMGNAVHTPISNMLPMIHPNDI

VWGGWDINNMNLGDAMKRAKVLDYDLQQQLYPHMKDIVPLPSVYFPDFIASNQNERANNV

LTGTKQEQMEQLRKDIRDFKSTNNLDKVIILWTANTERFASVEEGVNDRATTVLNSIKRD

EPEISASTVFAVASILEGCTFINGSPQNTFVPGVIELARQQRVFLAGDDFKSGQTKMKSV

LVDFLVSAGIKPVSIVSYNHLGNNDGKNLSAPNQFRSKEISKSNVVDDMVASNNILFEKD

EHPDHVVVIKYVPHVADSKRAMDEYTSEIFMGGKNTIVMHNTCEDSLLATPLIYDLVILG

ELCERITMGMEPWEAFHPVLSLLSYMLKAPLVPNGAPVVNALFAQRQAITNILRACGLAP

ENHMSL

[8] GAX27432.1

VQSSKCQYKSDAIIANFNYETTLVVVRPVETKLQFRTERKVPKLGVMLVGMGGNNGCTCI

AGAIANKLKVTWQTKEGEKKANYWGSVMLASTVKLGNDAVGNAVHTPISNMLPMVHPNDI

VWGGWDINNMNLGDAMKRAKVLDYDLQQQLYPHMKDIVPLPSVYFPDFIASNQNERANNV

LTGTKQEQMEQLRKDIRDFKATNNLDKVIILWTANTERFASVEEGVNDRATSVLNSIKRN

EPEISASTVFAVASILEGCTFINGSPQNTFVPGVVELARQQRVFLAGDDFKSGQTKMKSV

LVDFLVSAGIKPVSIVSYNHLGNNDGKNLSAPNQFRSKEISKSNVVDDMVASNNILFEKD

EHPDHVVVIKYVPHVADSKRAMDEYTSEIFMGGKNTIVMHNTCEDSLLATPLIYDLVILG

ELCERITMGTEPWEAFHPVLSMLSYMLKAPLVPNGAPVVNALFAQRQAITNVLRACGLAP

ENHMSL

[9] OEU16376.1

VRSDKCKYTEDTIISDFKYESTLVVVIPVETKMQFKTERAVPKVGVMLVGLGGNNGCTCI

AGAIANKLGMTWNTKEGEKKSNYWGSVMMSSTVKLGNDARGNSVFTPMKNMLPMVDPSQV

VWGGWDINSMNLGDAMRRSKVLDWDLQQKLYENMKDIVPLPSVYFPDFIAANQSERADHV

LTGTKQEQMEHIRKDISDFKAKHQLDKVILLWTANTERFACVEEGVNDTKENMLHSIQRD

EAEISASTIFAVASILEGCTFINGSPQNTFVPGVVDLARHKKVFIAGDDFKSGQTKMKSV

LVDFLVGAGIKPVSIVSYNHLGNNDGKNLSAPSQFRSKEISKSNVVDDMVESNRILFEED

EHPDHVVVIKHVPYVGDSKRALDEYTSEIFMGGVNTISMHNTCEDSLLATPLIYDLVILA

ELCDRISVGESEWEQFHPVLSLLSYMLKAPLVPNGAPVVNALFTQRQAIINVLRACGLGP

ENHMSL

[10] XP_002179168.1

VQSKNCQYNADTIISDYTYETTIVVVKPVKSKMKFRTERKVPKLGVMLVGLGGNNGCTCV

AGAIANKLGMTWETKEGKLKANYWGSVMMASTAKIGNDTHGNAVYTPLRNMLPMANPNEI

VWGGWDINGMNLGEAMKRSKVLDIDLQNQLYPHMKNITPLPSVYFSDFIAANQGERANNV

IKGTKQEQLDQLRNDIRNFKTNNGLDKVILLWTANTERFASIEEGVNDTADNLLESIMRG

EAEVSASTVFAVASILEGSTFINGSPQNTFVPGVLELARQHKVFIAGDDFKSGQTKMKSV

LVDFLVSAGIKPVSIVSYNHLGNNDGKNLSAPSQFRSKEISKSNVVDDMVASNRILFEED

EHPDHVVVIKYVPYVADSKRAMDEYTSEIFMGGKNTIVMHNTCEDSLLATPLIYDLVILG

ELCERITVGSEDWEAFHPVLSLLSYMLKAPLVPNGAPVVNALFTQREAVINVMRACGLGP

NNHMTL

[11] XP_012896216.1

VNAPNVTYTDDTITADYVYHTSEAVVTPRDEKYVFKTDRRIPKVGMMLIGWGGNNGTTVT

AGILANKHQITWHTKKGEMHPNYYGSISQSSTVKIGTVPGGD-LYIPFKNLLPLVNPNDL

VIGGWDISSMNLGDAMKRAGVYDYDFQQVMYPYMKDFKPLPSIYYPDYIAANQKDRADNV

IPGNKQVHLDTVRADMRRFKEENHLDKVIVLWTANTERYSEIIPGVNDTAENLLKSIRES

HSEVAPSTIFAVAAILEGNTYINGSPQNTFVPGVFELAEQHKTFIAGDDFKSGQTKIKSV

LVDYLVSAGIKPASIASYNHLGNNDGKNLSSPQQFRSKEISKSGVVDDMVASNGILYPNH

EHPDHLIVIKYIPFVGDSKRALDEYTSEIFNGGINTISMHNTCEDSLLAAPLMVDLVVLS

EMCERISLGSQGFERFNSVLSILSYLLKAPEVPRGTPVVNALSKQRECIINIFRACGLQP

DNHMML

[12] XP_012898387.1

VNASNVTYTDDTITADYVYHTSEAVVTPRDEKYVFKTDRRIPKVGMMLIGWGGNNGTTVT

AGILANKHQITWHTKKGEMHPNYYGSISQSSTVKIGTVPGGD-LYIPFKNLLPLVNPNDL

VIGGWDISSMNLGDAMKRAGVYDYDFQQVMYPYMKDFKPLPSIYYPDYIAANQKDRADNV

IPGNKQVHLDTVRADMRRFKEENHLDKVIVLWTANTERYSEIIPGVNDTAENLLKSIRES

HSEVAPSTIFAVAAILEGNTYINGSPQNTFVPGVFELAEQHKTFIAGDDFKSGQTKIKSV

LVDYLVSAGIKPASIASYNHLGNNDGKNLSSPQQFRSKEISKSGVVDDMVASNGILYPNH

EHPDHLIVIKYIPFVGDSKRALDEYTSEIFNGGINTISMHNTCEDSLLAAPLMVDLVVLS

EMCERISLGSQGFERFNSVLSILSYLLKAPEVPRGTPVVNALSKQRECIINIFRACGLQP

DNHMML

[13] GAX94616.1

VNSKNVTYTDDEIVSNYTYHVTKVVVTPVEEKYTLKTQRKVPKVGVMIIGLGGNNGSTLV

ASVLANKHNISWTTKEGVQQPNYFGSVTQASTVRLGTNAQGEGVFIPFNQLLPMVHPNDM

VIGGWDISSLNLGDAMKRAQVLDYDLQRQLYPMMKDIKPLPSIYYPDFIAANQADRADNV

LKGSKQENLEQIRKQIRDFKTSNGCDKVIVLWSANTERYSDIIEGVNDTSANLLESIKAG

EDEISPSTIFAVASILEGCSYINGSPQNTFVPGVLDLAEEKKIFVGGDDFKSGQTKMKSV

LVDFLVSAGIKPVSIVSYNHLGNNDGKNLSAPQQFRSKEISKSNVVDDMVASNQLLYKEG

EHPDHVVVIKYVPFVGDSKRALDEYTSKIFMNGTNTISMHNTCEDSLLATPLIIDLVVIC

ELAERIQLGNAEFERLHSVLSLLSYMLKAPLVPRGTPVVNALFAQRECIINLFRACGLPA

ESHMAL

[14] XP_008890663.1

VNSKNVVYTDDEITSQYTYTTTRVVATPVEEKFTFKTQRKIPKLGVMIVGLGGNNGSTLL

ASIIANKHHITWNTKEGVQEPNYFGSVTQASTVRLGTNANGEGVYIPFHNLLPMVSPNDL

VIGGWDISSLNMAEAMKRAMVLDHDLQRQLVPHLEKIKPLPSIYYPDFIAANQADRADNL

LKGSKQENLDAVRQQIRDFKQSNNLDKVIVLWSANTERFSDIVEGVNDTSANLLESIKAG

EAEISPSTIFAVASILEGCSYINGSPQNTFVPGVLDLAEEKKVFVGGDDFKSGQTKMKSV

LVDFLVSAGIKPTSIVSYNHLGNNDGKNLSAPQQFRSKEISKSNVVDDMVASNRLLYKEN

EHPDHVVVIKYVPFVGDSKRALDEYTSKIFMNGNNTISMHNTCEDSLLASPLILDLVLVC

EIAERITLGAKEFEHMHSVLSILSYMLKAPLVPRGTPVVNALFAQRECMINIFRACGLAP

ESHMLL

[15] XP_024579037.1

VNSPNVVYTEDEITSKYTYTTTRVVATPVEEKYTFKTQRKVPKVGVMIVGLGGNNGSTLV

ASVIANKHQITWNTKEGVQHPNYFGSITQASTVRLGTNANGEGVYIPFHNLLPMAAPNDL

VIGGWDISSLNMAEAMKRAQVLDHDLQRQLVPYLEKIKPLPSIYYPDFIAANQADRADNL

LKGSKQENLDALRQQIRDFKQNNGLDKVIVLWSANTERFSDIVTGINDTSANLLKSIQTN

EDEVSPSTIFAVASILEGCSYINGSPQNTFVPGVLDLAEEKKVFIGGDDFKSGQTKMKSV

LVDFLVSAGIKPTSIVSYNHLGNNDGKNLSAPQQFRSKEISKSNVVDDMVASNRLLYKEN

EHPDHVVVIKYVPFVGDSKRALDEYTSKIFMNGNNTISMHNTCEDSLLATPLILDLVLVC

ELAERITIGAKDFEHMHSVLSILSYMLKAPLVPRGTPVVNALFAQRECMINIFRACGLAP

ESHMLL

[16] KUF98217.1

VNSKNVVYTDDEITSQYTYTTTRVVATPVEEKFTFKTQRKIPKLGVMIVGLGGNNGSTLL

ASIIANKLHITWNTKEGVQEPNYFGSVTQASTVRLGTNANGEGVYIPFHNLLPMVSPNDL

VIGGWDISSLNMAEAMKRAMVLDHDLQRQLVPHLEKIKPLPSIYYPDFIAANQADRADNL

LKGSKQENLDAVRQQIRDFKQSNNLDKVIVLWSANTERFSDIVEGVNDTSANLLESIKAG

EAEISPSTIFAVASILEGCSYINGSPQNTFVPGVLDLAEEKKVFVGGDDFKSGQTKMKSV

LVDFLVSAGIKPTSIVSYNHLGNNDGKNLSAPQQFRSKEISKSNVVDDMVASNRLLYKEN

EHPDHVVVIKYVPFVGDSKRALDEYTSKIFMNGNNTISMHNTCEDSLLASPLILDLVLVC

EIAERITLGAKEFEHMHSVLSILSYMLKAPLVPRGTPVVNALFAQRECMINIFRACGLAP

ESHMLL

[17] OWZ08203.1

VNSKNVVYTDDEITSQYTYTTTRVVATPLEEKFTFKTQRKVPKLGVMIVGLGGNNGSTLL

ASILANKHHITWTTKEGVQEPNYFGSVTQASTVRLGTNANGEGVYIPFHNLLPMVSPNDL

VIGGWDISSLNMAEAMKRAEVLDYDLQRQLVPHLEKIKPLPSIYYPDFIAANQADRADNL

LKGSKQEQLDAVRQQIRDFKQSNGLDKVIVLWSANTERFSDIVEGVNDTSANLLESIKAS

EAEISPSTVFAVASILEGCSYINGSPQNTFVPGVLDLAEEKKVFVGGDDFKSGQTKMKSV

LVDFLVSAGIKPTSIVSYNHLGNNDGKNLSAPQQFRSKEISKSNVVDDMVASNRLLYKEN

EHPDHVVVIKYVPFVGDSKRALDEYTSKIFMNGNNTISMHNTCEDSLLASPLILDLVLVC

EIAERITLGASEFEHMHSVLSVLSYMLKAPLVPRGTPVVNALFAQRECMINIFRACGLAP

ESHMLL

[18] KUF75721.1

VNSKNVVYTDDEITSQYTYTTTRVVATPVEEKFTFKTQRKIPKLGVMIVGLGGNNGSTLL

ASIIANKLHITWNTKEGVQEPNYFGSVTQASTVRLGTNANGEGVYIPFHNLLPMVSPNDL

VIGGWDISSLNMAEAMKRAMVLDHDLQRQLVPHLEKIKPLPSIYYPDFIAANQADRADNL

LKGSKQENLDAVRQQIRDFKQSNNLDKVIVLWSANTERFSDIVEGVNDTSANLLESIKAG

EAEISPSTIFAVASILEGCSYINGSPQNTFVPGVLDLAEEKKVFVGGDDFKSGQTKMKSV

LVDFLVSAGIKPTSIVSYNHLGNNDGKNLSAPQQFRSKEISKSNVVDDMVASNRLLYKEN

EHPDHVVVIKYVPFVGDSKRALDEYTSKIFMNGNNTISMHNTCEDSLLASPLILDLVLVC

EXAERITLGAKEFEHMHSVLSILSYMLKAPLVPRGTPVVNALFAQRECMINIFRACGLAP

ESHMLL

[19] POM76882.1

VNSKNVVYTDDEITSQYTYTTTRVVATPVEEKFTFKTQRKVPKLGVMIVGLGGNNGSTLL

ASILANKHHITWTTKEGVQEPNYFGSVTQASTVRLGTNANGEGVYIPFHNLLPMVAPNDL

VIGGWDISSLNMAEAMKRAQVLDHDLQRQLVPHLEKIKPLPSIYYPDFIAANQADRADNL

LKGSKQEHLDAVRKQIRDFKQSNNLDKVIVLWSANTERFSDIVEGVNDTSANLLESIKAN

EAEVSPSTIFAVASILEGCSYINGSPQNTFVPGVLDLAEEKKIFVGGDDFKSGQTKMKSV

LVDFLVSAGIKPTSIVSYNHLGNNDGKNLSAPQQFRSKEISKSNVVDDMVASNRLLYKEN

EHPDHVVVIKYVPFVGDSKRALDEYTSKIFMNGNNTISMHNTCEDSLLASPLILDLVLIC

EIAERITLGAAEFEHMHSVLSILSYMLKAPLVPRGTPVVNALFAQRECMINIFRACGLAP

ESHMLL

[20] XP_002908320.1

VNSKNVVYTDDEITSQYTYTTTRVVATPVDEKFTFKTQRKIPKLGVMIVGLGGNNGSTLV

ASIIANKHHITWNTKEGVQEPNYFGSVTQASTVRLGTNANGEGVYIPFHNLLPMVSPNEL

VIGGWDISSLNMAEAMKRAMVLDHDLQRQLVPHLEKIKPLPSIYYPDFIAANQADRADNL

LKGSKQENLDAVRQQIRDFKQSNSLDKVIVLWSANTERFSDIVEGVNDTSANLLESIKAG

EAELSPSTIFAVASILEGCSYINGSPQNTFVPGVLDLAEEKKVFVGGDDFKSGQTKIKSV

LVDFLVSAGIKPTSIVSYNHLGNNDGKNLSAPQQFRSKEISKSNVVDDMVASNRLLYKEN

EHPDHVVVIKYVPFVGDSKRALDEYTSKIFMNGNNTISMHNTCEDSLLASPLILDLVLVC

ELAERITLGAAEYEHMHSVLSILSYMLKAPLVPRGTPVVNALFAQRECMINIFRACGLAP

ESHMLL

[21] RAW35630.1

VNSKNVVYTDDEITSQYTYTTTRVVATPLEEKFTFKTQRKVPKVGCMIVGLGGNNGSTLL

ASIIANKHHITWTTKEGVQEPNYFGSVTQASTVRLGTNANGEGVYIPFHNLLPMVSPNDL

VIGGWDISSLNMAEAMKRAMVLDHDLQRQLVPHLEKIKPLPSLYYPDFIAANQADRADNL

LKGSKQENLDAVRQQIRDFKQSNNLDKVIVLWSANTERFSDIVEGVNDTSANLLESIKAG

EAEISPSTIFAVASILEGCSYINGSPQNTFVPGVLDLAEEKKVFVGGDDFKSGQTKIKSV

LVDFLVSAGIKPTSIVSYNHLGNNDGKNLSAPQQFRSKEISKSNVVDDMVASNRLLYKEN

EHPDHVVVIKYVPFVGDSKRALDEYTSKIFMNGNNTISMHNTCEDSLLASPLILDLVLVC

ELAERITLGAAEFEHMHSVLSVLSYMLKAPLVPRGTPVVNALFAQRECMINIFRACGLAP

ESHMLL

[22] XP_009523578.1

VNSKNVVYTDAEITSQYTYTTTRVVATPLEEKYTFKTQRKVPKTGVMIVGLGGNNGSTLL

ASILANKHHITWTTKEGVQEPNYYGSVTQASTVRLGTNANGEGVYIPFHNLLPMVSPNDL

VIGGWDISSLNMAEAMKRAQVLDHDLQRQLVPYLEKIKPLPSIYYPDFIAANQADRADNL

LKGSKQENLDAVRKQIRDFKQSNGLDKVIVLWSANTERFSDIVEGVNDTSANLLESIKAG

EAEISPSTIFAVASILEGCSYINGSPQNTFVPGVLDLAEEKKIFVGGDDFKSGQTKMKSV

LVDFLVSAGIKPTSIVSYNHLGNNDGKNLSAPQQFRSKEISKSNVVDDMVASNRLLYKEN

EHPDHVVVIKYVPFVGDSKRALDEYTSKIFMNGNNTISMHNTCEDSLLASPLILDLVLVC

ELAERITLGASEFEHLHSVLSILSYMLKAPLVPRGTPVVNALFAQRECMINIFRACGLAP

ESHMLL

[23] XP_009821348.1

VNSKDVKYTESHIEANYTYQTSFVVVTPVSEQYLFKTNTKIPKLGIMIVGLGGNNGSTLV

AGVLANKLGLTWTDKDGEHKPDYFGSITQSSTVRLGTNAEGRGVYVPFKNMLPMVHPNDL

VIGGWDISAHNLADAMVRAKVLDYDLQRQLIPHLETITPLPSIYYPDFIAANQADRANNV

LTGSKQDNLNQIRQHIRDFKAKNSLDKVIVLWSANTERFSDIVEGVNDTADNLLEAIKNG

EDEVSPSTVFAVASILENTSYINGSPQNTFVPGAIELAERHNVFIGGDDFKSGQTKMKSV

LVDFLVGAGIKPVSIVSYNHLGNNDGKNLSAPQQFRSKEISKSNVVDDMVDSNRILFAED

EHPDHCVVIKYVPFVGDSKRALDEYTSKIFMNGTNTIAMHNTCEDSLLATPLILDLVIIC

ELAERIQIG-GKTERFHSVLSILSYLLKAPLVPRGTPIVNALFAQRECIVNILRACGLPA

ENYIQL

[24] RHY94259.1

VNSKDVKYTESHIEANYTYQTSFVVVTPVSEQYLFKTNTKIPKLGIMIVGLGGNNGSTLV

AGVLANKLGLTWTDKDGEHKPDYFGSITQSSTVRLGTNAEGRGVYVPFKNMLPMVHPNDL

VIGGWDISAHNLADAMVRAKVLDYDLQRQLIPHLETITPLPSIYYPDFIAANQADRANNV

LTGSKQDNLNQIRQHIRDFKAKNSLDKVIVLWSANTERFSDIVEGVNDTADNLLEAIKNG

EDEVSPSTVFAVASILENTSYINGSPQNTFVPGAIELAERHNVFIGGDDFKSGQTKMKSV

LVDFLVGAGIKPVSIVSYNHLGNNDGKNLSAPQQFRSKEISKSNVVDDMVDSNRILFAED

EHPDHCVVIKYVPFVGDSKRALDEYTSKIFMNGTNTIAMHNTCEDSLLATPLILDLVIIC

ELAERIQIG-GKVERFHSVLSILSYLLKAPLVPRGTPIVNALFAQRECIVNILRACGLPA

ENYIQL

[25] RLN67203.1

VNSKNVVYTDDEITSNYTYNTTRVVATPVEEKYTFKTQRKLPKLGVMIVGLGGNNGSTLV

ASTIANKHHITWTTKEGVQEPNYFGSVTQASTVRLGTNANGEGVYIPFHNLLPMVSPNDL

VIGGWDISSLNMAEAMKRAQVLDHDLQRQLVPHLEKIKPLPSIYYPDFIAANQADRADNL

LKGTKQENLDAVRAQIRDFKQSNGLDKVIVLWSANTERFSDIVEGVNDTTANLLESIKAG

EAEVSPSTIFAVASILEGCSYINGSPQNTFVPGVLDLAEEKKVFVGGDDFKSGQTKMKSV

LVDFLVSAGIKPTSIVSYNHLGNNDGKNLSAPQQFRSKEISKSNVVDDMVASNRLLYKEN

EHPDHVVVIKYVPFVGDSKRALDEYTSKIFMNGNNTISMHNTCEDSLLATPLILDLVLVC

ELAERITIGVAEYEHMHAILSILSYMLKAPLVPRGTPVVNALFAQRECMINIFRACGLPA

ESHMLL

[26] RLN20242.1

VNSKNVVYTDDEITSNYTYNTTRVVATPVEEKYTFKTQRKLPKLGVMIVGLGGNNGSTLV

ASTIANKHHITWTTKEGVQEPNYFGSVTQASTVRLGTNANGEGVYIPFHNLLPMVSPNDL

VIGGWDISSLNMAEAMKRAQVLDHDLQRQLVPHLEKIKPLPSIYYPDFIAANQADRADNL

IKGTKQENLDAVRAQIRDFKQSNGLDKVIVLWSANTERFSDIVEGVNDTTANLLESIKAG

EAEVSPSTIFAMASILEGCSYINGSPQNTFVPGVLDLAEEKKVFVGGDDFKSGQTKMKSV

LVDFLVSAGIKPTSIVSYNHLGNNDGKNLSAPQQFRSKEISKSNVVDDMVASNRLLYKEN

EHPDHVVVIKYVPFVGDSKRALDEYTSKIFMNGNNTISMHNTCEDSLLATPLILDLVLVC

ELAERITIGVAEYEHMHAILSILSYMLKAPLVPRGTPVVNALFAQRECMINVFRACGLPA

ESHMLL

[27] CCA14059.1

VESPNVQYTEQEIISQYAYNVSRVKVIPTSEKFTFRTQRKVPKVGAMIVGLGGNNGSTLV

ASILANKLGIQWNTKEGVQSPNYYGSITQASTVRLGTNSSGEGVFIPFSNILPMVHPNDL

VIGGWDISCMNLADAMKRAQVLEYDLQRQLIPHMEKMKPLPSIYYPDFIAANQSDRADNV

LTGSKQDNLEAIRNHIREFKKQHGLDKVIVLWSANTERYSDIVEGVNDTGKNLLESIKSG

EVEISPSTIFAVASILERCSYINGSPQNTFVPGVLDLAEEKSVFVGGDDFKSGQTKIKSV

LVDFLVSAGIKPVSIVSYNHLGNNDGKNLSAPQQFRSKEISKSNVVDDMVASNALLYKDG

EHPDHVVVIKYVPFVRDSKRAMDEYTSKIFMNGTNTIAMHNTCEDSLLATPLILDLVIVC

ELAERISINKSP-EKLHSILSLLSYMLKAPLVPRGTPVVNALFAQRECMVNIFRACGLSP

DNHMLL

[28] XP_008862724.1

VNSKDVKYTATHIEANYTYQTTYVVATPVAEQYLFKTSTKIPKLGVMIVGLGGNNGSTLV

AGVLANKLGITWSDKDGEHKPDYFGSITQSSTVRLGTNSEGRGVYVPFKNMLPMVNPNDL

VIGGWDISAHNLADAMVRAKVLDYDLQRQLIPHLEKITPLPSIYYPDFIAANQADRANNV

LTGSKQDNLNQIRQHIRDFKQKNSLDKVIVLWSANTERFSDIVEGVNDTADNLLEAIKNG

EDEVSPSTVFAVASILENTSYINGSPQNTFVPGAIELAERHKVFIGGDDFKSGQTKMKSV

LVDFLVGAGIKPVSIVSYNHLGNNDGKNLSAPQQFRSKEISKSNVVDDMVDSNRILFDKD

EHPDHCVVIKYVPFVGDSKRAMDEYTSKIFMNGTNTIAMHNTCEDSLLATPLILDLVIIC

ELAERIEIG-GSTERFHSVLSILSYLLKAPLVPRGTPIVNALFAQRECIVNILRAAGLPA

ENYIQL

[29] CCI44519.1

VESPNVQYTEQEIISQYAYNVSRVKVIPTSEKFTFKTQRKVPKVGAMIVGLGGNNGSTLV

ASILANKLGIQWNTKDGVQNPNYFGSVTQASTVRLGTNSSGEGVFIPFSNILPMVHPNDL

VIGGWDISCLNLAEAMKRAQVLEYDLQRQLIPHMEKIEPLPSIYYPDFIAANQSDRANNV

LTGSKQENLEAIRNHIREFKKEHELDKVIVLWSANTERYSDIIEGVNDTGKNLLESIKAS

EDEVSPSTVFAVASILEGCSYINGSPQNTFVPGVLDLAEQKSVFVGGDDFKSGQTKMKSV

LVDFLVSAGIKPVSIVSYNHLGNNDGKNLSAPQQFRSKEISKSNVVDDMVASNPLLYKDG

EHPDHVVVIKYVPFVRDSKRAMDEYTSKIFMNGTNTIAMHNTCEDSLLATPLILDLVIIC

ELAERISIN-RAAEKLHSVLSLLSYMLKAPLVPRGTPVVNALFAQRECMINIFRACGLPP

ENHMLL

[30] OQR85630.1

VNSPDVVYGGDKITTNYTYATTHFVATPIKEDYVFTTSTKVPKLGVMIVGLGGNNGSTLV

AGVLANKLGLSWTDKEGLHQADYFGSITQSSTVRLGTNNKGEGVYVPFKNMLPMVNPNDF

VIGGWDISGHNLADAMVRAKVLDYDLQRQLIPHMEKIKPLPSIYYPDFIAANQADRADNV

LQGSKQDNLEAIRTHIRDFKKNNGCDKVIVLWSANTERFSDIVEGVNDTADNILASIKAG

EDEVSPSSIFAVASILEGVSYINGSPQNTFVPGVVELAEKHNVFIGGDDFKSGQTKMKSV

LVDFLVGAGIKPVSIVSYNHLGNNDGKNLSAPQQFRSKEISKSNVVDDMVDSNRILFAEG

EHPDHCVVIKYVPFVGDSKRAMDEYTSKIFLNGTNTIVMHNTCEDSLLATPLILDLVIIC

ELAERIQIGAASPERFHSVLSILSYMLKAPLVPKGTPIVNALFAQRECIINILRACGLPA

ESYMQL

[31] XP_012201001.1

VNSKDVVYSAEAITTNYTYATTHFVATPVSEEYVITTSTKVPKLGVMIVGLGGNNGSTLV

AGVLANKLGLTWTDKDGLHKADYFGSVTQSSTVRLGTNNKGEGVYVPFKNMLPMVNPNDF

VIGGWDISGHNLADAMVRAKVLDYDLQRQLIPHMEKIKPLPSIYYPDFIAANQADRADNV

LQGSKQDNLETIRSHIREFKKSNGCDKVIVLWSANTERFSDIVEGVNDSAENILEAIKAG

EDEVSPSSVFAIASILEGASYINGSPQNTFVPGVVELAEKHKVFIGGDDFKSGQTKMKSV

LVDFLVGAGIKPVSIVSYNHLGNNDGKNLSAPQQFRSKEISKSNVVDDMVDSNRILFNEG

EHPDHCVVIKYVPFVGDSKRAMDEYTSKIFLNGTNTIVMHNTCEDSLLATPLILDLVIIC

ELAERIQIGAAEAERFHSVLSILSYMLKAPLVPKGTPIVNALFAQRECIINILRACGLPA

ENYMQL

[32] XP_008605672.1

VNSKDVVYSAEAITTNYTYATTHFVATPVSEEYVITTSTKVPKLGVMIVGLGGNNGSTLV

AGVLANKLGLTWTDKEGLHKADYFGSVTQSSTVRLGTNNKGEGVYVPFKNMLPMVNPNDF

VIGGWDISGHNLADAMVRAKVLDYDLQRQLIPHMEKIKPLPSIYYPDFIAANQADRADNV

LQGSKQDNLETIRSHIRDFKKSNGCDKVIVLWSANTERFSDIVEGVNDSAENILEAIKAG

EDEVSPSSVFAIASILEGASYINGSPQNTFVPGVVELAEKHKVFIGGDDFKSGQTKMKSV

LVDFLVGAGIKPVSIVSYNHLGNNDGKNLSAPQQFRSKEISKSNVVDDMVDSNRILFNEG

EHPDHCVVIKYVPFVGDSKRAMDEYTSKIFLNGTNTIVMHNTCEDSLLATPLILDLVIIC

ELAERIQIGAAEAERFHSVLSILSYMLKAPLVPKGTPIVNALFAQRECIINILRACGLPA

ENYMQL

[33] OQS05457.1

VNSKDVVYTAEHITTNYTYSTTHFVATPVHEEYVFKTSTKVPKLGVMIVGLGGNNGSTLV

AGVLANKLGLKWQDKEGEHKADYFGSVTQSSTVRLGTNNKGEGVYVPFKNMLPMVNPNDF

VIGGWDISGHNLADAMVRAKVLDYDLQRQLIPHMQKIKPLPSIYYPDFIAANQADRADNV

LNGSKQDNLEAIRGHIRDFKKNNGCDKVIVLWSANTERFSDIVEGVNDTAENLLASIKAG

EDEVSPSSVFAVASILEGASYINGSPQNTFVPGVVELAEKHKIFIGGDDFKSGQTKMKSV

LVDFLVGAGIKPVSIVSYNHLGNNDGKNLSAPQQFRSKEISKSNVVDDMVDSNRILFKEG

EHPDHCVVIKYVPFVGDSKRAMDEYTSKIFLNGTNTIVMHNTCEDSLLATPLILDLVIIC

ELAERIQIGNASPERFHSVLSILSYMLKAPLVPKGTPIVNALFAQRECIINILRACGMDP

REWLSF

[34] RLN68240.1

------------------------------------------------------------

------------------------------------------------------------

------------------------------------------------------------

------------RQQIRDFKQSNGLDKVIVLWSANTERFSDIVEGVNDTSANLLESIKAG

EAEVSPSTVFAVASILEGCSYINGSPQNTFVPGVLDLAEEKKVFVGGDDFKSGQTKIKSV

LVDFLVSAGIKPTSIVSYNHLGNNDGKNLSAPQQFRSKEISKSNVVDDMVASNRLLYKEG

EHPDHVVVIKYVPFVGDSKRALDEYTSKIFMNGNNTISMHNTCEDSLLATPLILDLIIVC

ELAERVTIGAAGFEHLHSILSILSYMLKAPLVPRGTPVVNALFAQRECMINVFRACGLPA

ENHMLL

[35] RLN58161.1

------------------------------------------------------------

------------------------------------------------------------

------------------------------------------------------------

------------RQQIRDFKQSNGLDKVIVLWSANTERFSDIVEGVNDTSANLLESIKAG

EAEVSPSSVFAVASILEGCSYINGSPQNTFVPGVLDLAEEKKVFVGGDDFKSGQTKIKSV

LVDFLVSAGIKPTSIVSYNHLGNNDGKNLSAPQQFRSKEISKSNVVDDMVASNRLLYKEG

EHPDHVVVIKYVPFVGDSKRALDEYTSKIFMNGNNTISMHNTCEDSLLATPLILDLLIVC

ELAERVTIGAAGFEHLHSILSILSYMLKAPLVPRGTPVVNALFAQRECMINVFRACGLPA

ENHMLL

[36] POM73461.1

------------------------------------------------------------

-------------------EPNYFGSVTQASTVRLGTNANGEGVGFFVPDFSRAAAPNSL

ASGGWDISSLNMAEAMKRAQVLDHDLQRQLVPHLEKIKPLPSIYYPDFIAANQADRADNL

LKGSKQEHLDAVRKQIRDFKQSNNLDKVIVLWSANTERFSDIVEGVNDTSANLLESIKAN

EAEVSPSTIFAVASILEGCSYINGSPQNTFVPGVLDLAEEKKVFVGGDDFKSGQTKMKSV

LVDFLVSAGIKPTSIVSYNHLGNNDGKNLSAPQQFRSKEISKSNVVDDMVASNRLLYKEN

EHPDHVVVIKYVPFVGDSKRALDEYTSKIFMNGNNTISMHNTCEDSLLASPLILDLVLIC

EIAERITLGAAEFEHMHSVLSILSYMLKAPLVPRGTPVVNALFAQRECMINIFRACGLAP

ESHMLL

[37] GBG32238.1

VDSPNVQYTEDEIASTYSYASTNVKAAPEQKEVQFRTQRKVGKVGAMIVGLGGNNGSTLI

AGIMANKLGLEWRTKEGVQKPNYYGSVTMASTIRLGTDESGNDIFVPFNSLVPMVHPNDL

VIGGWDISKMNLGDAMRRAKVLDVSLQDQLYPHMKDIVPLPSLYFPDFIAANQSDRADNV

LTGSKQEQLEQIKADIEAFKKDNDLDKVIVLWSANTERFSEIQEGVNDSMSNLMQAIEED

EEEISPSTLFAVAAIETGSAYINGSPQNTFVPGVIELAQERNVYIGGDDFKSGQTKFKSA

LVDFLVSAGIAPRSIVSYNHLGNNDGKNLSAPKQFRSKEISKSNVVDDMVQSNGILYKPG

EHPDHCVVIKYVPYVGDSKRAMDEYTCEIFMAGKQTIVSHNTCEDSLLATPLILDLVIVT

ELLQRVTFGEADHHGFHSVLSLLSYMLKAPLTPPGTPVVNSLFTQREAIVNFLRAFGLPP

ESHMSL

[38] EWM29092.1

VESPDVTYTGDEILAKYTYETVVIKAIPTKTLFTFKTKRHVPKTGLMLVGWGGNNGTTTT

AGILANRMKLSWRTKEGERHANYFGSLTQASTVRLGNDKFGNSVYVPFHRLLPMLHPNDL

VIGGWDISKQDLGDAMRRAQVLDAGLQDQLYPHMKALQPLPSIYFPDFIAANQADRADNV

LTGSKQEQLDAVRAHIRDFKASHSLEKVIVLWTANTERFAAVEEGNNDTAENLLAAIERE

EEELSPSTLFAVASILEGCSYINGSPQNTFVPGVIDLAQQRGVYVGGDDFKSGQTKMKSV

LVDFLVSAGIKPTSIVSYNHLGNNDGKNLSAPQTFRSKEISKSNVVDDMVASNRLLYAED

EHPDHVVVIKYVPCVGDSKRAMDEYTSEIFMGGTNTIVMHNTCEDSLLATPLIYDLVVLT

EMAERITVGDGTFESFASVLSLLSYLIKAPLVPPGTPVVNALFAQRQCIVNIFRACGLPP

DNNMLL

;

END;

BEGIN NOTES;

TEXT TAXON=1 TEXT='contig SL_25748.1_384_1991_- [Saccharina latissima]

';

TEXT TAXON=2 TEXT='contig SL_25960.2_333_2118_- [Saccharina latissima]

';

TEXT TAXON=3 TEXT='scaffold6694|size3610:531-3396(-) [Saccharina japonica]

';

TEXT TAXON=4 TEXT='Inositol-3-phosphate synthase [Ectocarpus siliculosus]

';

TEXT TAXON=5 TEXT='myo-inositol-1-phosphate synthase-like protein, partial [Thalassiosira pseudonana CCMP1335]

';

TEXT TAXON=6 TEXT='hypothetical protein THAOC_12648 [Thalassiosira oceanica]

';

TEXT TAXON=7 TEXT='myo-inositol-1-phosphate synthase [Fistulifera solaris]

';

TEXT TAXON=8 TEXT='myo-inositol-1-phosphate synthase [Fistulifera solaris]

';

TEXT TAXON=9 TEXT='MI-1-P synthase [Fragilariopsis cylindrus CCMP1102]

';

TEXT TAXON=10 TEXT='predicted protein [Phaeodactylum tricornutum CCAP 1055/1]

';

TEXT TAXON=11 TEXT='uncharacterized protein [Blastocystis hominis]

';

TEXT TAXON=12 TEXT='uncharacterized protein [Blastocystis hominis]

';

TEXT TAXON=13 TEXT='inositol-3-phosphate synthase, putative [Pythium insidiosum]

';

TEXT TAXON=14 TEXT='hypothetical protein PPTG_00853 [Phytophthora parasitica INRA-310]

';

TEXT TAXON=15 TEXT='inositol-3-phosphate synthase [Plasmopara halstedii]

';

TEXT TAXON=16 TEXT='40S ribosomal protein S11 [Phytophthora nicotianae]

';

TEXT TAXON=17 TEXT='Inositol-3-phosphate synthase [Phytophthora megakarya]

';

TEXT TAXON=18 TEXT='Inositol-3-phosphate synthase [Phytophthora nicotianae]

';

TEXT TAXON=19 TEXT='Inositol-3-phosphate synthase [Phytophthora palmivora var. palmivora]

';

TEXT TAXON=20 TEXT='inositol-3-phosphate synthase, putative [Phytophthora infestans T30-4]

';

TEXT TAXON=21 TEXT='Inositol-3-phosphate synthase [Phytophthora cactorum]

';

TEXT TAXON=22 TEXT='hypothetical protein PHYSODRAFT_350632 [Phytophthora sojae]

';

TEXT TAXON=23 TEXT='hypothetical protein H257_00382 [Aphanomyces astaci]

';

TEXT TAXON=24 TEXT='hypothetical protein DYB35_003447 [Aphanomyces astaci]

';

TEXT TAXON=25 TEXT='hypothetical protein BBP00_00001765 [Phytophthora kernoviae]

';

TEXT TAXON=26 TEXT='hypothetical protein BBI17_001977 [Phytophthora kernoviae]

';

TEXT TAXON=27 TEXT='PREDICTED: inositol3phosphate synthase 1like putative [Albugo laibachii Nc14]

';

TEXT TAXON=28 TEXT='hypothetical protein H310_01403 [Aphanomyces invadans]

';

TEXT TAXON=29 TEXT='unnamed protein product [Albugo candida]

';

TEXT TAXON=30 TEXT='inositol-3-phosphate synthase [Achlya hypogyna]

';

TEXT TAXON=31 TEXT='inositol-3-phosphate synthase [Saprolegnia parasitica CBS 223.65]

';

TEXT TAXON=32 TEXT='inositol-3-phosphate synthase [Saprolegnia diclina VS20]

';

TEXT TAXON=33 TEXT='inositol-3-phosphate synthase [Thraustotheca clavata]

';

TEXT TAXON=34 TEXT='hypothetical protein BBJ28_00011071, partial [Nothophytophthora sp. Chile5]

';

TEXT TAXON=35 TEXT='hypothetical protein BBJ28_00022214, partial [Nothophytophthora sp. Chile5]

';

TEXT TAXON=36 TEXT='Inositol-3-phosphate synthase, partial [Phytophthora palmivora var. palmivora]

';

TEXT TAXON=37 TEXT='Inositol-3-phosphate synthase [Hondaea fermentalgiana]

';

TEXT TAXON=38 TEXT='inositol-3-phosphate synthase [Nannochloropsis gaditana]

';

END;

1. ML phylogenetic tree of stramenopile INO1 protein sequences. The tree was built using PhyML^[3]^ (LG model; aLRT branch support; model-given amino-acid frequencies; optimized across-site rate variation; best of NNI and SPR tree search). *S. latissima* INO1 contigs are well-supported within the Phaeophyceae (brown algal) clade. The non sex-biased copy SL_25748.1 is sister to the *S. japonica* INO1. The female-biased copy SL_25960.2 is more divergent. It should be noted that following Lipinska et al. (2017)^[4]^, the *S. japonica* genome sequence derives from a male gametophyte strain.


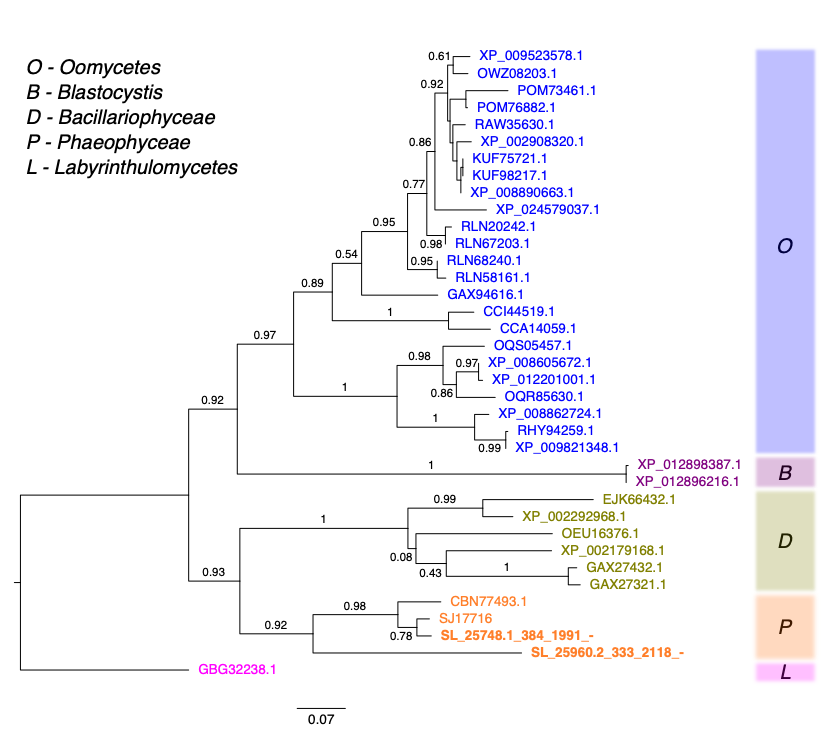


References

[1]. Edgar RC. MUSCLE: multiple sequence alignment with high accuracy and high throughput. Nucleic Acids Res. 2004 Mar 19;32(5):1792–7.

[2]. Castresana J. Selection of conserved blocks from multiple alignments for their use in phylogenetic analysis. Mol Biol Evol. 2000 Apr;17(4):540–52.

[3]. Guindon S, Dufayard J-F, Lefort V, Anisimova M, Hordijk W, Gascuel O. New algorithms and methods to estimate maximum-likelihood phylogenies: assessing the performance of PhyML 3.0. Syst Biol. 2010 May;59(3):307–21.

[4]. Lipinska AP, Toda NRT, Heesch S, Peters AF, Cock JM, Coelho SM. Multiple gene movements into and out of haploid sex chromosomes. Genome Biol. 2017 Dec 8;18(1):104.
